# DNA methylation of METTL7A gene body regulates its transcriptional level in thyroid cancer

**DOI:** 10.18632/oncotarget.16147

**Published:** 2017-03-13

**Authors:** Shasha Zhou, Yihang Shen, Min Zheng, Linlin Wang, Raymond Che, Wanning Hu, Pin Li

**Affiliations:** ^1^ Department of Endocrinology, Shanghai Children's Hospital, Shanghai Jiao Tong University, Shanghai, 200040, People's Republic of China; ^2^ Institute of Biomedical Sciences, Shanghai Medical College of Fudan University, Shanghai, 200032, People's Republic of China; ^3^ Department of Oncology of Tangshan People's Hospital & Tangshan Cancer Hospital, North China University of Science and Technology, Tangshan, Hebei, 063001, People's Republic of China; ^4^ Department of Oncology, Jinan Central Hospital Affiliated to Shandong University, Jinan, Shandong, 250013, People's Republic of China; ^5^ Department of Radiation Oncology, Shandong Cancer Hospital and Institute, Jinan, Shandong, 250117, People's Republic of China; ^6^ University of Hawaii Cancer Center, University of Hawaii, Honolulu, Hawaii, 96814, USA

**Keywords:** DNA methylation, gene body, EZH2, METTL7A, thyroid cancer

## Abstract

DNA methylation is the best-studied epigenetic mechanism for regulating gene transcription and maintaining genome stability. Current research progress of transcriptional regulation by DNA methylation mostly focuses on promoter region where hypomethylated CpG islands are present transcriptional activity, as hypermethylated CpG islands generally result in gene repression. Recently, the DNA methylation patterns across the gene body (intragenic methylation) have increasingly attracted attention towards their role in transcriptional regulation and efficiency, due to the improvement of numerous genome-wide DNA methylation profiling studies. However, the function and mechanism of gene body methylation is still unclear. In this study, we revealed that the methylation level of METTL7A, a seldom studied gene, was downregulated in thyroid cancer compared to normal thyroid cells *in vivo* and *in vitro*. Moreover, we determined the methylation level of one CpG site at the exon of the METTL7A gene body impacted the transcriptional activity. Through generating a mutation of this CpG site (CG to CC) of METTL7A exogenous vector artificially *in vitro*, we observed higher RNA polymerase II recruitment and a declined enrichment of methyl-CpG binding protein-2 in gene body of METTL7A, in papillary thryoid cancer cells (BCPAP) compared to normal thryoid cells. Finally, we revealed that EZH2, a subunit of polycomb repressor complex 2, dominant in thyroid cancer, might be responsible for regulating gene body methylation of METTL7A. Our study depicted the DNA methylation patterns and the transcriptional regulatory mechanism of the gene body in thyroid cancer. Furthermore, this study provides new insight into potential future avenues, for therapies targeting cancer.

## INTRODUCTION

Currently, DNA methylation, the most widely studied epigenetic way, is a covalent modification in which the 5 position of pyrimidine ring in cytosine base of the dinucleotide sequence 5’CpG3’ (Cytosine-phosphate-guanine) is appended to a methyl group by DNA methyltransferases. The methylated cytosine residing in the major groove of DNA helix can be recognized by methyl-CpG-binding proteins (MBPs), which can further recruit NuRD repressor complex, Sin3A and HDAC to remodel chromosomal structure, repel RNA polymerase and finally cause transcriptional suppression [1–4]. Most studies reveal that abnormal DNA methylation within the promoter regions of genes causes transcriptional silencing in disease processes such as cancer [5].

Thyroid cancer is one of the most common types of malignancy in the endocrine system. In the past few decades, the prevalence of thyroid cancer has been appreciably increasing around the world [6]. There are four main types of thyroid cancer, papillary and mixed papillary/follicular (80%), follicular and Hurthle cell (15%), medullary (3%) and anaplastic (2%). Although, the molecular pathogenesis of thyroid cancer is not fully clear. In recent years, research has gradually incorporated a broader understanding on DNA methylation alteration associated with multiple canonical pathway dysregulation [7] as well as thyroid cancer subtypes classification for early diagnosis [8]. Most studies focus only on the gene promoter regions where the methylation alteration is the cause for silencing of a particular gene that can be interpreted as an important event for physiology of the cells. However, the synergetic effect for gene expression by the methylation of promoter regions, and non-promoter regions, such as gene body, UTR and distal, remains unknown. With the development of genome-wide DNA methylation analysis, the increasingly number of studies primarily focus on DNA methylation of non-promoter regions. In several researches of invertebrates and plants, gene body methylation is put forward as a potential mechanism of RNA alternative splicing regulation [9], and a tight correlation with some higher biological functions and individual behaviors [10, 11] as well as the gene family expansion and functional diversification in evolution [12]. In vertebrates, actively transcribed genes are associated with hypomethylation of promoter region and oppositely increased DNA methylation levels of gene body, which indicates a possible new universal function of DNA methylation [13–15]. Nonetheless, the epigenetic regulatory roles of the transcriptional elongation process within the gene body have not been fully illuminated, with regards to cancer.

Herein, we investigated the mRNA profiles (GDS1732, GDS1665) of thyroid cancer, obtained through public databases. Additionally, we observed methyltransferase like 7A (METTL7A, also called AAM-B) and found this gene was down-regulated in thyroid cancers compared to control. Finally, we focused on the DNA methylation change of METTL7A in thyroid cancer *in vivo* and *in vitro* in order to provide insight into the epigenetic regulatory mechanism of gene body methylation during tumorigenesis.

## RESULTS

### The relationship between mRNA and the methylation level of METTL7A in human thyroid cancer

**Figure 1:**
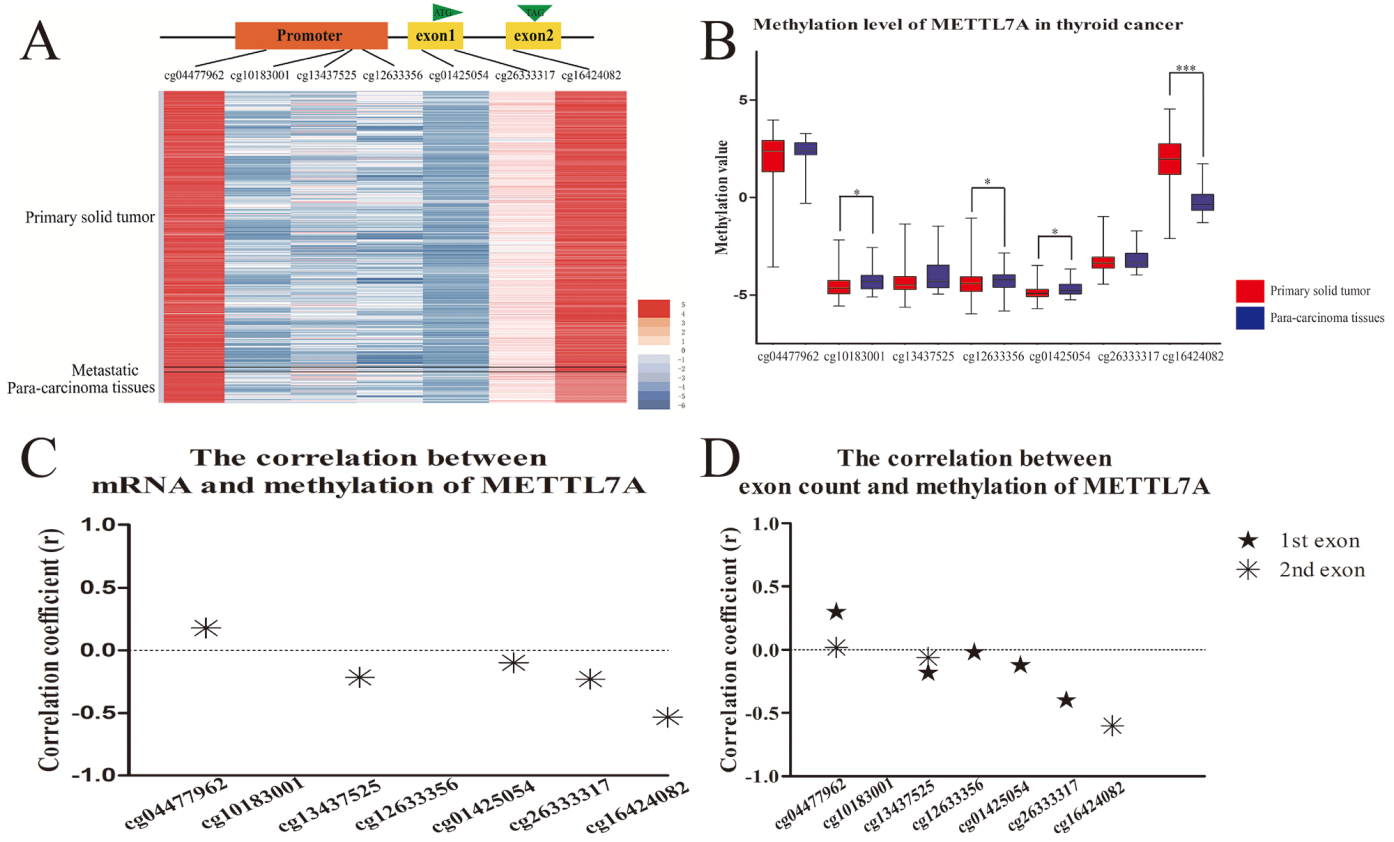
DNA methylation level of METTL7A in thyroid cancers *in vivo* **(A)** The heatmap of DNA methylation patterns of METTL7A in thyroid cancer patients from TCGA database. The methylation level from high to low represents red to blue. Black lines divide all the samples into three parts of primary tumor, metastatic tumor and control tissues. **(B)** Analysis of methylation level of METTL7A between thyroid cancer and control tissues. METTL7A related methylation probe names from promoter to gene body were listed on the horizontal axis. **(C)** The correlation between mRNA and mthylation level of each probe of METTL7A. The correlations of METTL7A mRNA level and methylation value of each probe were calculated separately using Pearson analysis. Positive and negative correlation coefficients which had statistical significance (p<0.05) were plotted by asterisk above and below 0 respectively. **(D)** The correlation between exon count and mthylation level of each probe of METTL7A. The correlations of METTL7A first and second exon count and methylation value of each probe were calculated separately using Spearman analysis. Positive and negative correlation coefficients which had statistical significance were plotted by stars above and below 0 respectively.

The presence of METTL7A down-regulation in our preliminary analysis of the microarray profiles of human papillary thyroid cancer (GDS1732 and GDS1665), in comparison to normal thyroid tissue suggested that METTL7A might be repressed in tumorigenesis. However, the role of METTL7A in transcriptional regulation in thyroid cancer is unknown. In this study, we investigated the DNA methylation and RNA landscape of human thyroid cancer *in vivo* from The Cancer Genome Atlas (TCGA) databases with a central focus on METTL7A. The DNA methylation level of METTL7A in tumors was observed to be distinct from para-carcinoma tissues (Figure 1A). The significant difference of methylation level in CpG sites of METTL7A between primary solid thyroid tumors and para-carcinoma tissues was observed in the promoter regions (cg10183001, cg12633356 and cg01425054) and particularly remarkable in the gene body (cg16424082) (Figure 1B). Nevertheless, the promoter regions displayed higher methylation in normal tissues while gene body showed higher methylation level in the tumor group. Furthermore, the transcriptional expression of METTL7A mined from mRNA profiles of the identical samples using TCGA databases had a negative correlation with methylation level of most of CpG sites, including gene body methylation in the comparison between normal and tumor samples (Figure 1C). Additionally, RNA sequencing data showed that the counts of first and second exon of METTL7A were also negatively correlated with the gene body methylation (cg16424082) (Figure 1D). These results suggest that firstly the abnormal DNA methylation alteration of METTL7A occurred in thyroid cancer and secondly the CpG loci especially in gene body (cg16424082) affect the transcriptional level of METTL7A.

### Transcriptional regulation of METTL7A by gene body methylation in thyroid cancer cell lines

**Figure 2:**
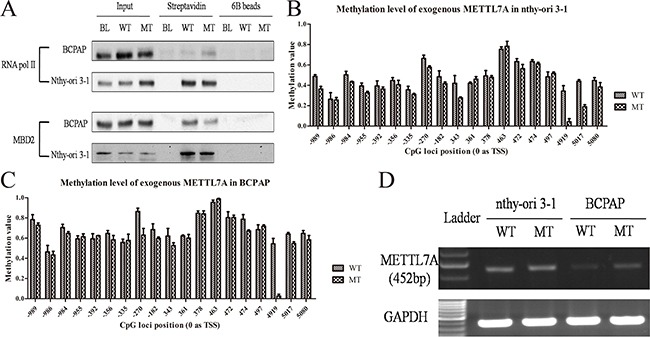
Gene body methylation of METTL7A in thryoid cancer cell lines **(A)** The enrichment of RNA pol II and MBD2 in gene body of exogenous linear METTL7A with wild type (WT) and mutant (MT) +4919 CpG site in BCPAP and nthy-ori 3-1 thyroid cell lines. **(B, C)** Methylation level of the exogenous METTL7A template with wild type and mutant +4919 CpG site in nthy-ori 3-1 and BCPAP cell lines. **(D)** The transcriptional level from exogenous METTL7A with wild type (WT) and mutant (MT) in nthy-ori 3-1 and BCPAP cell lines.

Currently, it is well acknowledged that DNA methylation mediated transcriptional regulation primarily revolves around its role at the promoter regions. However, understanding of methylation patterns across the non-promoter, such as the gene body for transcriptional regulation and efficiency still remains equivocal. In this study, we observed the methylation alteration of single CpG site of +4919 (0 as TSS) included in this methylation probe (cg16424082) showed more notable sensitivity than other CpG sites or islands at promoter regions and might play a crucial role in METTL7A exon elongation. Therefore, to follow through, we mainly focused on DNA methylated regulation of this CpG site of +4919 in thyroid cancers, which is located at the second exon of METTL7A, nearly 200bp prior to the termination codon. We cloned the full length of METTL7A genomic DNA and conducted mutagenesis PCR to destroy the CpG site of +4919. This was achieved via the substitution of 541-543 TCG with TCC, whilst encoding the equal serine (TCG/TCC). The linear DNA of METTL7A was amplified from the plasmid, labeled biotin and incubated with lysis of normal thyroid follicular epithelial cell line (nthy-ori 3-1) or papillary thyroid cancer cell line (BCPAP), respectively. Cells were then harvested by streptavidin beads and DNA methylation levels were investigated as well as the enrichment of RNA pol II and MBD2 of the exogenous template. Interestingly, the presence of declining MBD2 whereas increased RNA pol II enrichment in CpG mutant METTL7A template compared to the wild type one was observed in thyroid cancer cells but not in normal cells (Figure 2A). Meanwhile, pyrosequencing exhibited the loss of methylation at +4919 mutated CpG site and slightly reduced methylation level at promoter regions in mutant template compared to wild type METTL7A template in both thyroid cancer and normal cells (Figure 2B, 2C). And the methylation level of endogenous METTL7A in BCPAP and another cell line TPC-1 were also validated that DNA methylation modification of METTL7A in papillary thyroid cancer were consistent even if the different genetic background (Supplementary Figure 1). Consistently, using the specific primers to eliminate the background interruption from endogenous METTL7A transcripts, the mutant METTL7A transcript level of exogenous template were observed to be more highly expressed than wild type, although this was only in thyroid cancer cells (Figure 2D). Collectively, our results determine that gene body methylation affect METTL7A transcriptional initiation and silencing in thyroid cancer.

### Abnormal enrichment of EZH2 responsible for METTL7A methylation alteration in thyroid cancer

**Figure 3:**
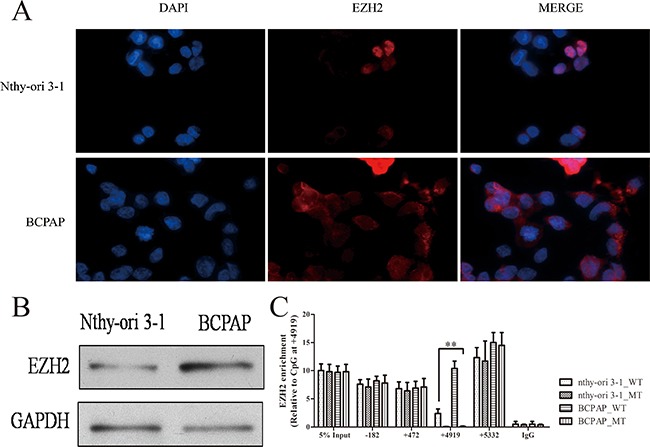
EZH2 expression and location in thryoid cancer cells **(A)** The location of EZH2 protein in nthy-ori 3-1 and BCPAP cell lines. The nthy-ori 3-1 and BCPAP cells were fixed and counterstained with DAPI (Blue) and EZH2 antibody (Green). Immunofluorescence images were processed using Olympus BX51 fluorescence microscope. **(B)** The protein expression of EZH2 was investigated in nthy-ori 3-1 and BCPAP cells using western blot (GAPDH as a loading control). **(C)** EZH2 enrichment in exogenous METTL7A with wild type (WT) and mutant (MT) in nthy-ori 3-1 and BCPAP cell lines. -182, +472, +4919, +5332 represent promoter, first exon, second exon and 3’UTR region of METTL7A.

Although gene body methylation has been revealed to play a crucial role in METTL7A transcriptional regulation, the enrichment of RNA pol II and MBD2 to modulate METTL7A transcriptional change only occurs in thyroid cancer cells, however, the mechanism behind this is still unknown. We suspected that the regulatory protein profiles binding to the CpG site of METTL7A may be altered between cancer and non-cancerous cells. Therefore, by harvesting streptavidin after incubation within the cell suspension of nthy-ori 3-1 or BCPAP cells, the wild type exogenous template was conducted by blue staining (Supplementary Figure 2) and EZH2 was identified as one of the different patterns using Mass Spectrometry. EZH2 has been acknowledged as a histone methyltransferase catalytic subunit, which comprises part of a polycomb repressor complex (PRC2) and is reported to silence tumor suppressor genes when upregulated in cancers [16]. Therefore, to verify this identification of EZH2 in our study, we detected the expression of EZH2 in nthy-ori 3-1 and BCPAP cells using an immunofluorescence assay and western blot (Figure 3A, 3B), and observed that EZH2 was expressed higher in thyroid tumor cells compared to normal cells. ChIP-assay was conducted to investigate the interaction between EZH2 and exogenous METTL7A gene with wild type and mutant CpG site of 4919, including the promoter (-182), first exon (+472), second exon (+4919) and 3’UTR (+5332). It was notable that the enrichment of EZH2 was substantially higher, especially at the gene body region containing the +4919 CpG site in thyroid cancer compared to normal cells (Figure 3C). Interestingly, consistent with blue staining, we validated the loss of EZH2 enrichment in mutant exogenous METTL7A template compared to wild type template in cancer cell line, which indicated that METTL7A had a special methylation motif (central at +4919 CpG site) recognized by EZH2 at the gene body. Taken together, we determined that EZH2 may play an important role in gene body methylation regulation.

Overall, we revealed that EZH2 interacted with the CpG site at the gene body of METTL7A and was responsible for MBD2 recruitment as well as RNA pol II rejection, which leads to METTL7A silencing in thyroid cancer.

## DISCUSSION

METTL7A is reported as an integral membrane protein anchored into the endoplasmic reticulum membrane, to recruit cellular proteins for lipid droplet formation [17, 18]. Moreover, the intermediate region of METTL7A is classified as a putative function of S-adenosylmethionine-dependent methyltransferase. However, currently there is little experimental evidence to support this claim, as there are no related studies to METTL7A in cancer. Despite this, microarray profiles give us a clue that METTL7A is a differentially expressed during thyroid tumorigenesis. In addition, we confirmed that METTL7A mRNA is downregulated in thyroid cancer *in vivo*. METTL7A was considered to function as the common lipid metabolism and functional organelles production [19]. Therefore, we speculated that the decline of METTL7A may lead to tumorigenesis due to the disorder of lipid metabolism but need to be demonstrated in further study.

In this study, we mainly focused on gene body methylation and transcriptional regulation of METTL7A in thyroid cancer. In most cases, detecting methylation of the CpG sites or islands only at the promoter, or the region near TSS is highly sensitive and desirable to provide disease diagnosis and prognosis. With the technological advancement in genome-scale mapping of methylation, to evaluate DNA methylation signature in other different genomic contexts, such as gene body, 3’UTR, the regulatory elements or repeat sequences become possible. For gene body, CpG sites are extensively methylated although poorly distributed. Gene body methylation is often described as a tissue or cancer specific landscape [20], without the association to gene repression [13]. The presence of methylation at the second exon of METTL7A was inversely expressed with the promoter region between normal and cancer tissues indicated from our data (Figure 1B). This suggests that gene body methylation may play an alternative role that originally thought. With the validation of downregulated METTL7A mRNA expression in thyroid cancer, the gene body methylation of +4919 CpG site support the negative correlation at the transcriptional level. Whereas the methylation of the promoter region exhibited in negligible significance between normal and tumor tissues *in vivo* (Figure 1B), which implicates that gene body methylation exerts two probable ways for transcriptional regulation. Potentially, gene body methylation may impact transcriptional elongation and RNA alternative splicing [21, 22]. This is thought to be the case as the methylated exons are favored for nucleosome occupancy [23], which pauses or blocks the movement of RNA pol II. Conversely, the other possibility is an assumption called alternative promoter usage [20]. Most genes have at least two TSSs, whereby the subsequent sites are within the gene body of the transcriptional units of the upstream promoters. Furthermore, to block transcriptional elongation, the downstream promoter must have a strong CpG site or island compared to any prior promoter regions. The result of this leads to an apparent discordance between methylation and expression [24]. As METTL7A has no additional RNA variance, and only the transcripts of the second exon not first exon were obviously negative correlated with methylation level of gene body in thyroid cancer (Figure 1D). We speculate that the methylation in the second exon is likely a regulator transcriptional elongation and termination.

To figure out the potential function of gene body methylation during tumorigenesis, we generated a mutation of +4919 CpG site at the second exon and observed apparent change of RNA pol II and MBD2 enrichment (Figure 2A), which reflects that DNA methylation of gene body may also recruit methyl-binding proteins for gene silencing. Demethylation of a methylated cytosine in the coding sequence is a major cause of C to T transition mutations. Generally, once C to T mutation occurs, most triplet codes combinations encode for an alternative amino acid. Therefore, these types of mutations in germline and somatic cells can often lead to disease [25]. To demonstrate this, we destroyed specific CpG sites, whilst still maintaining the identical serine for translation. Subsequently, we observed the loss ability of being methylated in the cytosine (Figure 2B, 2C) whereby the mRNA alteration derived from the mutant template (Figure 2D), which may interpret that encoding DNA mutation may lead to disease occurrence via not only the dysfunctional proteins but also the disorder transcriptional control.

Interestingly, the presence of alternative transcriptional regulation upon wild type and mutant METTL7A is only observed in thyroid cancer cells but not in normal cells, which implies that cancer specific DNA methylation signatures of CpG sites or islands in the gene body participate in tumor programming. Previous study revealed that gene body methylation may particularly impact certain types of genes as an opposite way by Dnmt3b during carcinogenesis or DNA methylation inhibitors induction [26], which inspires us to investigate histone modification and try to figure out the different response to DNA methylation among the different areas of METTL7A. Here we reveal that EZH2, the functional enzymatic component of PRC2, which is responsible for addition of methyl groups to histone H3 at lysine 27 (H3K27), displays a particularly high recruitment towards METTL7A gene body in cancer cells (Supplementary Figure 2, Figure 3C). The differential expression and enrichment of EZH2 in cancer, compared to normal cells may suggest that the PRC2 complex plays an essential role in the selection and recognition of methylated nucleotide substrates. Additionally, our results also suggests that EZH2 is involved in the recruitment of methylation binding proteins or enzymes towards the preferential CpG sites. Moreover, although EZH2 is known to catalyze H3K27 methylation, our results also suggest that this particular histone modification may be not required for the interaction between EZH2 and methylated nucleotides. This is supported our findings, as our exogenous METTL7A template represents a naked linear DNA chain without any packaged nucleosome or chromatin structure. Finally, our study showed that EZH2 is extraordinarily upregulated in cancer cells, although the mechanism for this is unclear and further research is required. However, we do provide evidence that the alteration of DNA methylation patterns in disease states may be resulted from the disordered function of epigenetic modification enzymes.

Collectively, we expect that accumulating knowledge from understanding gene body methylation will provide valuable information in the future, especially to develop effective tools for DNA methylation targeted therapy in cancer prevention as well as therapy.

## MATERIALS AND METHODS

### Bioinformatic analysis

The DNA methylation assay (450 K), mRNA profiles, RNA sequence and clinical information of 449 primary thyroid carcinoma samples with 56 para-carcinoma tissues (level 3) were obtained from The Cancer Genome Atlas (TCGA) data portal, downloaded on April 8th 2016. The methylation values of METTL7A were gathered and converted into M-values by $\text{M}=\log_{2}\frac{\beta }{1-\beta }$ in compliance with a normal distribution, as described [27]. Student's t-test was used to analyze the methylation differences between thyroid cancer and control. The Pearson and Spearman correlation were used for analysis between the DNA methylation level and mRNA (converted into log2) or exon counts, respectively.

### Cell culture, chemicals and antibodies

The papillary thyroid cancer cell lines of BCPAP, TPC-1 and thyroid follicular epithelial cell line of nthy-ori 3-1 were obtained from the American Type Culture Collection (ATCC) and maintained in RPMI 1640 medium supplemented with 10% FBS at 37°C in 5% CO_2_ (v/v). Antibodies against RNA polymerase II (RNA pol II), MBD2 and EZH2 were purchased from Santa Cruz. TA cloning kit and Streptavidin CL-6B agarose beads were purchased from Thermo Fisher.

### Methylation assay

Genomic DNA was harvested using phenol-chloroform extraction. 2 μg DNA was treated with bisulfite using EpiTect Bisulfite Kit (Qiagen) and PCR was conducted. Pyrosequencing was then conducted, following the instruction of PyroMark PCR Kit (Qiagen). Three independent experiments were repeated for each sample. Primers for bisulfite sequences in this study were listed in Table 1.

**Table 1:** The primers for bisulfite sequence, ChiP-qPCR and molecular cloning in this study are listed. The thymines or adenines with lowercase letters represent the transformed cytosines after bisulfite treatment

Bisulfite sequence	Primer sequence	Tm(°C)	Length(bp)
-989, -986, -984, -955	F: 5’-AtATTGGTTtAGTttAGAAAGGt-3’	45	130
	R: 5’-CTTTCTATTtATCCCAaaTC -3’		
-392, -356, -335, -270, -182	F: 5’-TGGtAGtAtTtTTtTGGTGTGG-3’	48	287
	R: 5’-aaCCAATTTTaCCTAATAa-3’		
+343, +361, +378, +463, +472, +474, +497	F: 5’-GATtAAGAGtATTGtAGAGAAt-3’	50	210
	R: 5’-CTAACTAaTCCTCCTCACACCC-3’		
+4917, +5017, +5080	F: 5’-GGAGtATGTGGtAGtTGAGTG-3’	52	223
	R: 5’-CTCACACTATTTCACAaCATATCC-3’		
LINE-1	F: 5’-TTTTGAGTTAGGTGTGGGATATA-3’	55	188
	R: 5’-AAAATCAAAAAATTCCCTTTC-3’		
ChIP assay (-182)	F: 5’-CTTATGAAAACTAAGCTGAATCG-3’	52	83
	R: 5’-GCTGTGCTGGAAAGTCA-3’		
ChIP assay (+472)	F: 5’-TGGTGCTGTGCTCTGT-’3	55	97
	R: 5’-CTAACTAGTCCTCCTCACACCC-’3		
ChIP assay (+4909)	F: 5’-GCATGTGGCAGCTGAGTGTTCG-3’	60	66
	R: 5’-TGCCAGGCAGGATCCAGGACT-3’		
ChIP assay (+5332)	F: 5’- TCACTGCAACCTCCACCTC -’3	45	108
	R: 5’- CTA AAAATACAAATTAGC -’3		
METTL7A full length	F: 5’-GATTTATTCTGAGCCAAATATGAG-3’	48	8650
	R: 5’-TAAACCATATTTCTGTTCCTAAC-3’		
50bp deletion	F: 5’-TCAGCCTCCTGAGTAGCTGGATGGTGTTTCCC-3’	55	-
	R:5’-GGGAAACACCATCCAGCTACTCAGGAGGCTGA-3’		
Mutagenesis PCR	F: 5’-CATGTGGCAGCTGAGTGTTCCACTTGGAATTACTTCTGGC-3’	55	-
	R: 5’-GCCAGAAGTAATTCCAAGTCCAACACTCAGCTGCCACATG-3’		
METTL7A testing	F: 5’-AAAGAGTTTTGCTCTTGT-3’	60	452
	R: 5’- AATTAGCCGGCGTGGTGA-3’		
GAPDH	F: 5’-ATATGATTCCACCCATG-3	60	332
	R: 5’-GTGCTAAGCAGTTGGTGGT-3’		

### Plasmids

METTL7A full length DNA (8.7 kb) was cloned from nthy-ori 3-1 cell line and conducted into TOPO TA vector (Invitrogen). We deleted the 50 bp of noncoding region (+5681˜+5730) at the second exon and designed the testing primers here to distinguish the endogenous METTL7A pre-message RNA (Table 1). Mutation of +4919 CpG site (CG to CC) was generated by mutagenesis PCR. Primers were listed in Table 1. METTL7A linear DNA was amplified based on the vectors of normal and mutated template using biotin labeled dCTP. 5 μg PCR products were digested by Dpn I to remove the template and purified for the further experiments.

### Immunoprecipitation and immunoblotting

IP or ChIP assay were performed as described [28, 29]. Briefly, 5μg exogenous METTL7A linear DNA was incubated with 1ml suspension of nthy-ori 3-1 or BCPAP cells at 37°C for 3 h, then harvested by streptavidin beads or blank beads A agarose. The immunoprecipitants were washed using the appropriate buffer. And extracted proteins for RNA pol II and MBD2 were detected by western blot. The transfer-ready membrane was blocked in PBS containing 5% nonfat milk and 0.1% Tween-20, followed by incubation with primary antibody of the appropriate dilution. The secondary antibodies used were horseradish peroxidase-conjugated anti-rabbit (1:5000).

For ChIP assay, cells were sonicated and incubated with antibodies specific for either EZH2 or IgG then were extracted DNA and detected the promoter, gene body and 3’UTR regions of METTL7A gene, where the primer sets for qPCR were designed to encompass approximately 200 bp (Table 1). The Ct value was analyzed to calculate enrichment using delta-delta methods.

### Blue staining

After running, the SDS-PAGE gel was rinsed with water, stained using SimplyBlue™ Safe Stain (Novex) for 15 min and changed to new stain and incubated overnight then washed, using fresh water several times until the clear bands were displayed without background.

### Immunofluorescence assay

BCPAP and nthy-ori 3-1 cells were transplanted into 8 mm teflon printed black diagnostic slides (Immuno-Cell) overnight, and fixed within 3.7% formaldehyde for 15 min at 4°C next day and quenched using 0.125M glycine for 10 min. Following this, samples were washing using pre chilled PBS for 5 min × 3 times, cells were permeabilized using 0.2% Triton-100 for 5 min in room temperature, and incubated with 3% horse serum for 30 min, then primary antibody (diluted appropriately in 0.5% horse serum) overnight at 4°C. Samples were washed using pre chilled PBS for 5 min × 3 times, cells were incubated with goat anti-rabbit second antibody (Alexa Fluor 594) (Abcam) for 1 h in a cool and dark environment. Samples were washed in pre chilled PBS 5 min × 3 times. Cells were stained by 800mM DAPI and natural withering, then mounting medium was added before attaching the cover glass, sealed slides with nail polish, cells were observed under microscope.

### Statistical analysis

All data are presented as means ± standard deviations for three independent experiments. The differences in values were analyzed using Student's t-test. Statistical significance was established when the *p* value was less than 0.05 (*, *p* < 0.05; **, *p* < 0.01).

## SUPPLEMENTARY MATERIALS FIGURES AND TABLES


