# Supplementary material for: DNA methylation of METTL7A gene body regulates its transcriptional level in thyroid cancer
Source: Oncotarget. 2017 Mar 13;8(21):34652–60. doi: 10.18632/oncotarget.16147 (PMC5470999; doi:10.18632/oncotarget.16147)
Supplement: Supplementary file 1 [file oncotarget-08-34652-s001.pdf]

DNA methylation of METTL7A gene body regulates its transcriptional level in thyroid cancer

SUPPLEMENTARY FIGURES

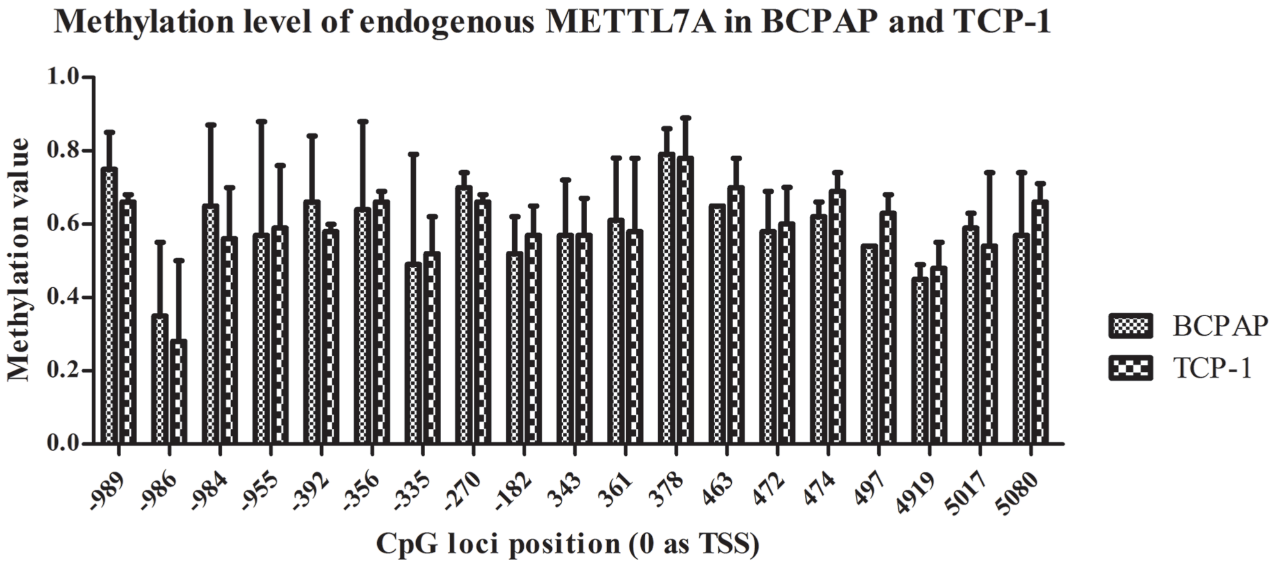

Supplementary Figure 1: Methylation level of endogenous genomic METTL7A in BCPAP and TPC-1 cell lines. The data of METTL7A in two cell lines is normalized by methylation level of LINE-1, which is thought as a stable methylated region and can represent the overall methylation level of genomic DNA.

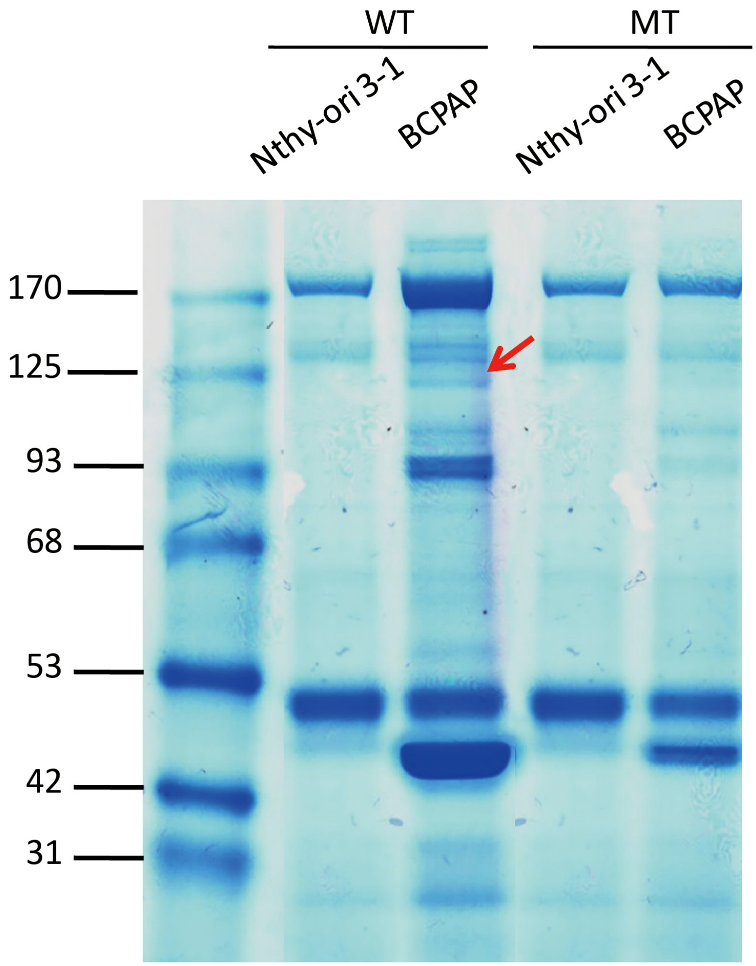

Supplementary Figure 2: The blue staining of the protein patterns of wild type and mutant METTL7A template in Nthy-ori 3-1 and BCPAP cells.
